# Supplementary material for: Identifying communities from multiplex biological networks
Source: PeerJ. 2015 Dec 22;3:e1525. doi: 10.7717/peerj.1525 (PMC4690346; doi:10.7717/peerj.1525)
Supplement: Supplemental Information 1 [file peerj-03-1525-s001.pdf]

Supplementary material: Identifying Communities from Multiplex Biological Networks

Gilles Didier[1,*], **Christine Brun**[2,3], **and Anaïs Baudot**[1,*]

[1] *Aix-Marseille Université, CNRS, Centrale Marseille, I2M UMR7373, Marseille, France*
[2] *TAGC, INSERM U1090, Aix-Marseille Université, Marseille, France*
[3] *CNRS, Marseille, France*
[*] *Corresponding authors: gilles.didier@univ-amu.fr, anais.baudot@univ-amu.fr*

1. SUPPLEMENTARY TABLES

The 4 biological networks were constructed from:

- PPI: PSICQUIC portal[1] and the CCSB Interactome database [2]
- Co-Expression: RNA-Seq data downloaded from the Human Protein Atlas (http://www.proteinatlas.org).Spearman correlations of expression values were computed between all genes based on FPKM values in 27 tissues and 44 cell lines, and correlation ≥ 0.7 were selected as interactions.
- 5 pathway databases: Biocarta (http://www.biocarta.com), Spike [3], Kegg [4], PID [5], Reactome [6] constructed using the R package *graphite* [7].
- Complexes: Corum database [8] using a matrix model.

For detailed protocol and options, the markdown file to generate biological networks is available at GitHub (https://github.com/gilles-didier/MolTi). Data were fetched and networks created in May, 2015.

Table S1. The Biological Networks, number of nodes, number of edges and network densities

Network	Number of nodes	Number of edges	density
Pathways	8 839	166 761	0.004
PPI	12 110	60 669	0.001
Co-expression	9 912	1 107 547	0.023
Complexes	2 528	36 762	0.012
Total (unique)	17 003	1 338 086	0.009

2. SUPPLEMENTARY FIGURES

A. Simulations and comparisons with GenLouvain

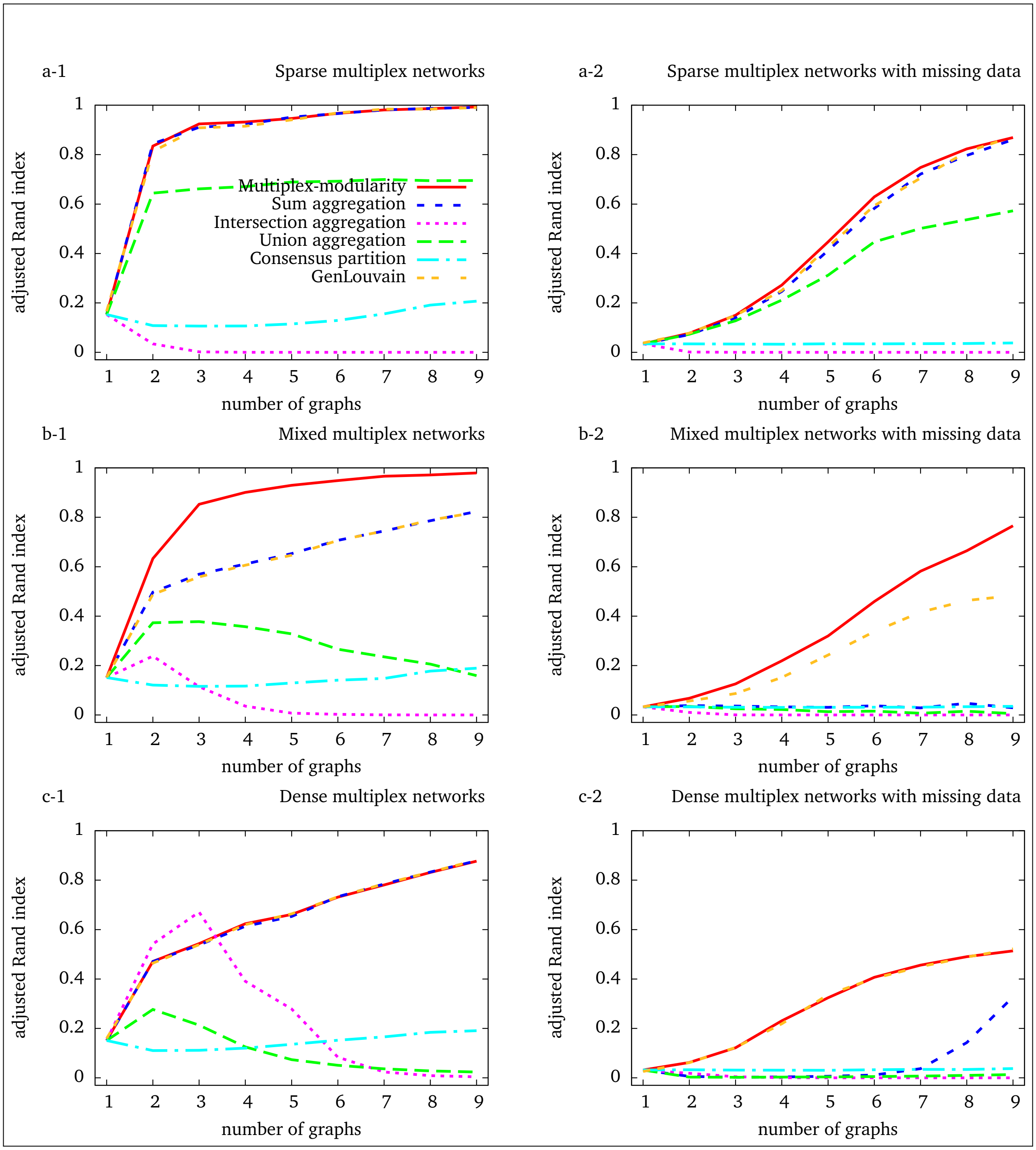

Fig. S1. Adjusted Rand indexes between the reference community structure used to generate the random multiplex networks and the communities detected by the different approaches. The communities are detected by optimizing the multiplex-modularity or by optimizing the classical modularity applied to networks aggregated via the intersection, union or sum of the graphs composing the multiplex networks or by the GenLouvain approach. The multiplex networks contain from 1 to 9 graph layers. The indexes are averaged over 100 random multiplex networks of 1 000 vertices and 20 balanced communities. Sparse (resp. dense) multiplex networks are simulated with 0.1/0.01 (resp. 0.5/0.2) internal/external edge probability matrix. Mixed multiplex networks are simulated by uniformly sampling among these two matrices. Each vertex is withdrawn from each graph with a probability 0.5 to generate missing data.

We extended the Figure 1 of the main manuscript to compare our multiplex-modularity approach with the general GenLouvain approach [9]. We applied GenLouvain using the "Categorical Multi-slice Matrix" procedure (as in example 3 of http://netwiki.amath.unc.edu/GenLouvain/GenLouvain with resolution parameter $\alpha = 1$ and inter-slice coupling parameter $\omega = 1$. As our own Louvain implementation is not randomized, we call GenLouvain with the "forces index-ordered" option (see the GenLouvain help). The source code of the corresponding simulation procedure (including the calls to GenLouvain), is available at gituhub (https://github.com/gilles-didier/MolTi) in the src/ folder (TestThreadGenLouvain.c). Without missing data, the results obtained with GenLouvain are close to those of the Sum-aggregation approach. This behavior was expected from the formula of the "multislice modularity" of [9] (with the particular parameters we used).

B. Biological networks and the resolution parameter

B.1. Community numbers, mean and median sizes, for different value of the resolution parameter

The number of communities (Figure S2), their mean (Figure S3) and median (Figure S4) sizes obtained after clustering. The classical Louvain algorithm that optimizes the network modularity [10] was directly applied to the multiplex networks aggregated through their Union or Sum. The algorithm was further adapted to optimize the Multiplex-modularity as the sum of the individual network modularities of the multiplex networks

We obtained from 87 to 1 634 communities for the Sum aggregation, and from 69 to 911 communities for the Multiplex-modularity approach, for $\gamma = 1$ and $\gamma = 15$, respectively (Supplementary Fig. S2). Module sizes are diverse, from 195 to 15 proteins per community on average for the Sum aggregation, or from 246 to 19 proteins per community for the Multiplex-modularity approach, for $\gamma = 1$ and $\gamma = 15$, respectively (Supplementary Fig. S3). Interestingly, the median sizes of the communities obtained after partitioning the largest networks remain very low (Supplementary Fig. S4). For instance, for the Sum aggregation partition, the community median sizes is of 2 proteins per module, for $\gamma = 1$ to $\gamma = 15$, respectively. This means that half of the detected communities contains less than 2 proteins and would be tough to interpret biologically.

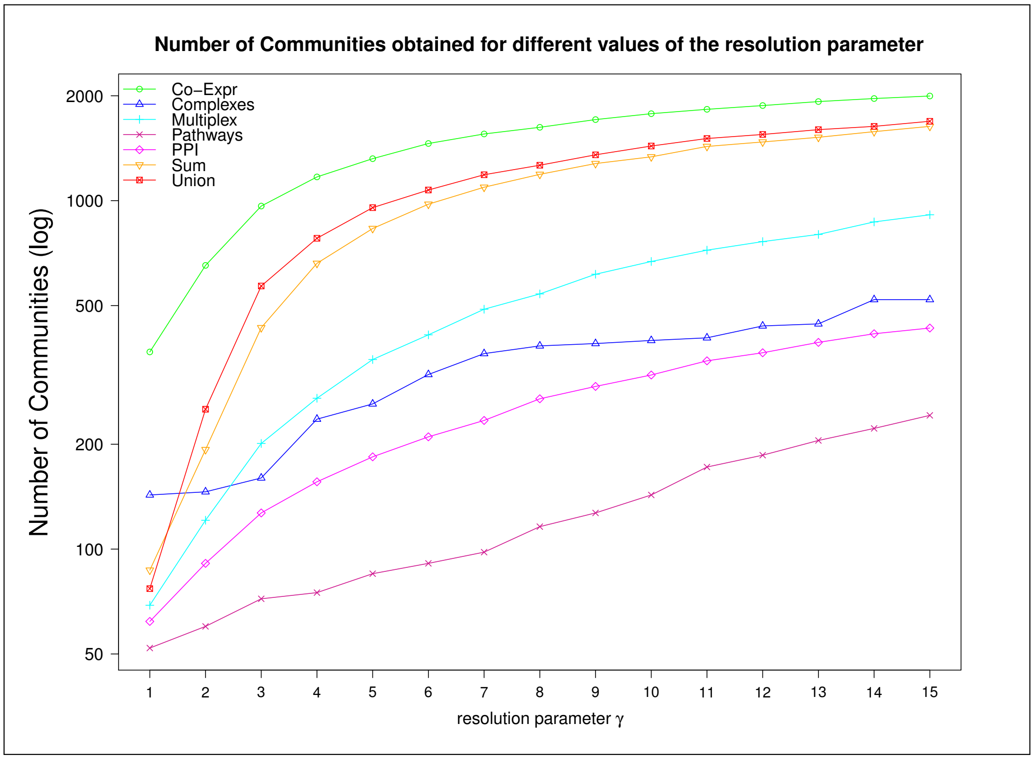

Fig. S2. Number of communities obtained with resolution parameter values ranging from γ=1 to γ=15, for the 4 individual networks, their Sum and Union aggregations, and the Multiplex-modularity approach.

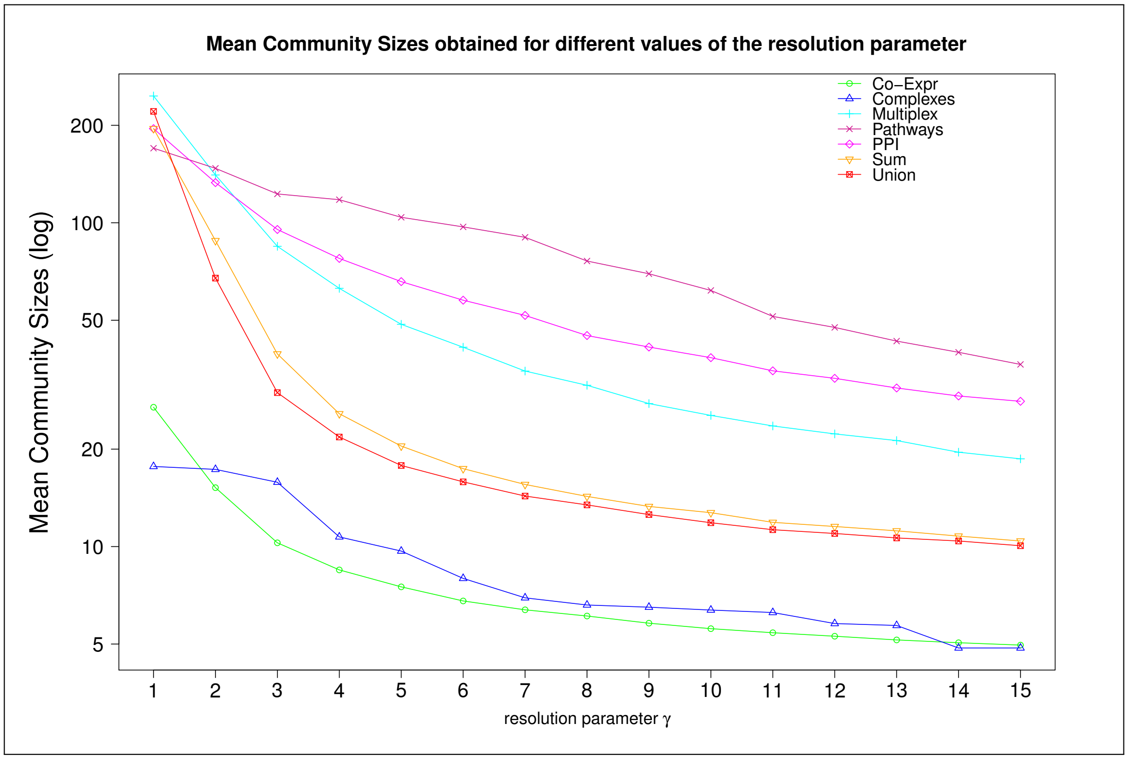

Fig. S3. Mean of the communities sizes obtained with resolution parameter values ranging from γ=1 to γ=15, for the 4 individual networks, their Sum and Union aggregations, and the Multiplex-modularity approach.

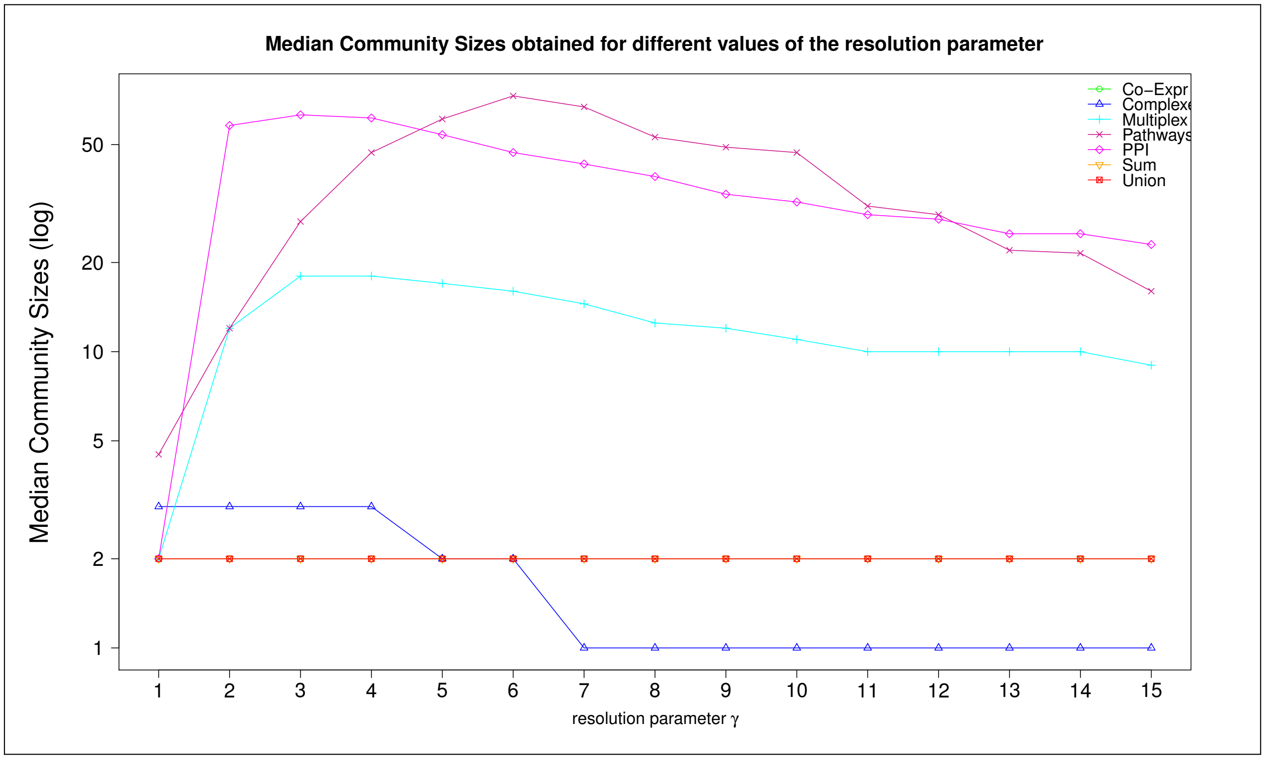

Fig. S4. Median of the communities sizes obtained with resolution parameter values ranging from γ=1 to γ=15, for the 4 individual networks, their Sum and Union aggregations, and the Multiplex-modularity approach. The Sum and Union curves are overlapping.

B.2. Distribution of Community sizes

Distribution of community sizes (i.e. number of nodes in each community) obtained after partitioning the 4 individual networks, their Sum and Union aggregations, and with the Multiplex-modularity approach, with a resolution parameter γ=5.

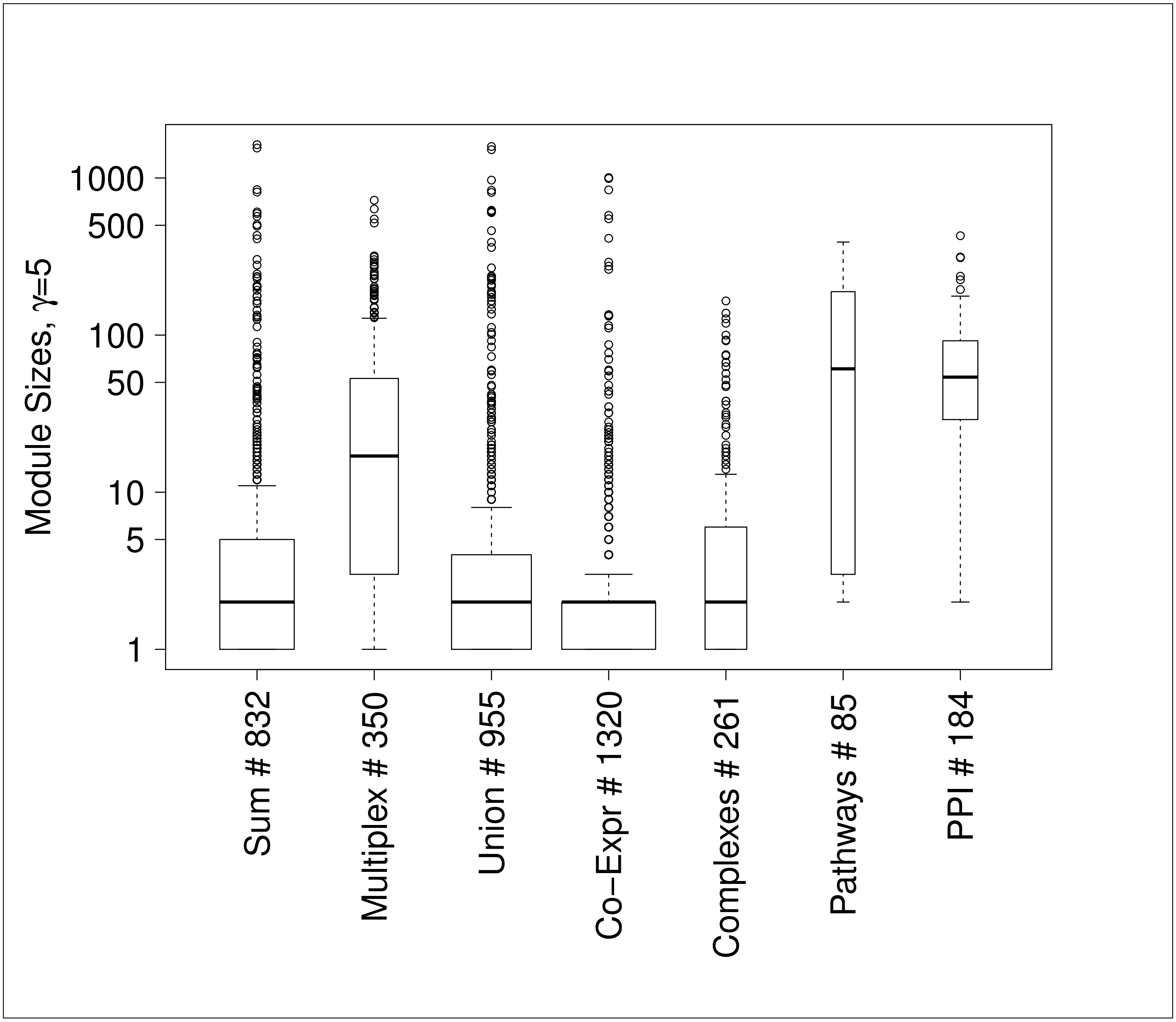

Fig. S5. Community size distributions for each of the partitions. The boxplot width are proportional to the number of communities obtained in each partition.

3. SUPPLEMENTARY CODE

A. Software

The *MolTi* software allows identifying communities from multiplex networks, and annotate the obtained clusters. A standalone user-friendly version of the software is available at Github (https://github.com/gilles-didier/MolTi). *MolTi* performs both clustering and annotation enrichment tests from multiplex networks, and allow an easy exploration of the results.

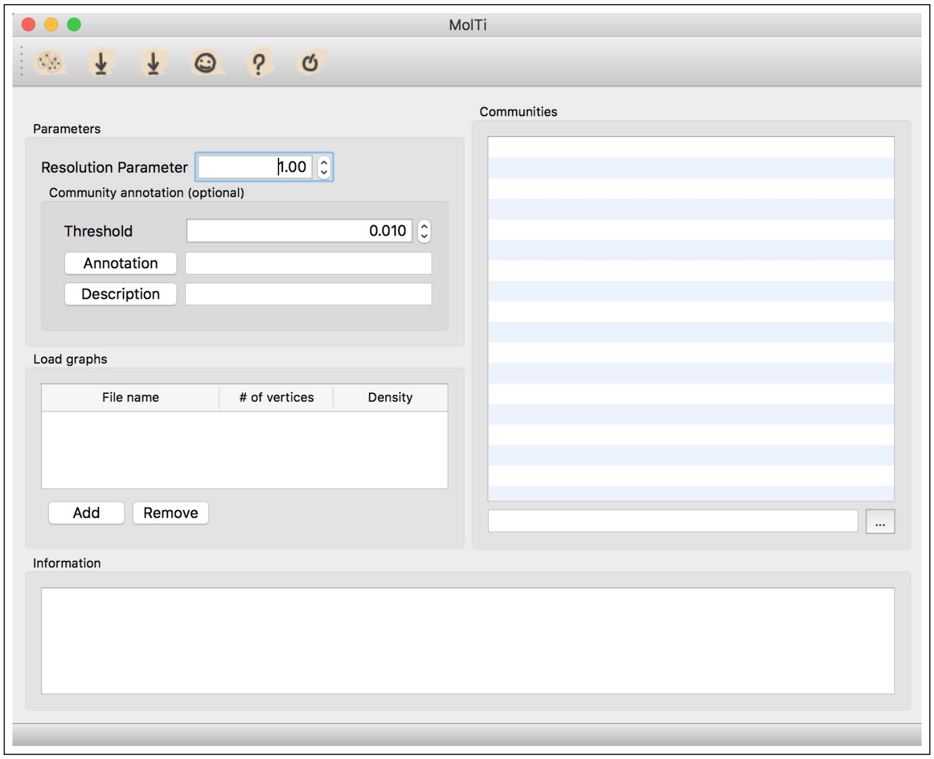

Fig. S6. Screenshot of the MolTi graphical interface

The source code of the *MolTi* software, *molti-console* (clustering), *bonf* (annotation), together with the code for *test* (simulation) is also available at GitHub, as well as examples of input and output files.

- *molti-console* takes as input several graph files in the simple "ncol" format (one interaction per line, the 2 interactors being separated by a tab), from which it detect communities by maximizing their Multiplex-modularity. It returns a file containing the community partition. Option '-p' sets the resolution parameter and Option '-s' provides the community partitions of all the individual graphs and that of their sum and union graphs (for comparison purpose).

```
usage: molti-console [options] <input file1> <input file2> ...

options are
-o <file name>      set the output file name prefix
-p <real number>    set the Newman modularity resolution parameter
-s                  compute partition on each graph individually and on the sum graph
-h                  display help
```

- *bonf* computes the annotation enrichment with an exact Fisher test and Bonferroni correction for multiple-testing, for all the communities of a given partition. It takes as input an annotation "ontology" file and a partition file (partition file may be in Clust'N'See [**?**] format/option '-f c' or in flat format, i.e. a line per community/option '-f f'). It returns a file listing all the communities significantly (i.e. with a corrected exact Fisher test lower than the value provided with option 'threshold') enriched in at least one ontology term (all the ontology terms significantly represented in a class are displayed below this one). The option '-o' allows providing a file containing descriptions of the ontology terms, which will be displayed in the output file.

```
usage: bonf [options] <annotation file> <partition file> [<output file>]

options are
-o <ontology descriptions file>     load ontology terms descriptions
-t <real number>                    set the threshold
```

```
-f c or f                              indicate the partition format
-h                                     display help
```

- *test* first simulates random multiplex networks with `g` vertices and from `1` to `t` layers with a balanced community structure of `c` communities (`i` simulations for each number of layers). It next detects communities by using aggregation and multiplex-modularity approaches and computes the adjusted Rand index (and the normalized mutual information) between the reference community structure and the detected one. It writes a '.csv' file containing the means and the standard deviations of the adjusted Rand indexes for each method, with the following format:
  - Column 1: number of layers
  - Column 2 and 3: mean and standard deviation obtained with the multiplex-modularity approach
  - Column 4 and 5: mean and standard deviation obtained with the sum-aggregation approach
  - Column 6 and 7: mean and standard deviation obtained with the intersection
  - Column 6 and 7: mean and standard deviation obtained with the union aggregation approach.

```
usage: test <options> <output file name>

Options:
-g <number>set the number of vertices of the random graphs
-t <number>set the max number of random graphs
-c <number>set the number of classes
-i <number>set the number of iterations
-p <prob intra> <prob inter>add a new pair of probas
-a  <number>set the modularity parameter
-h display help message
```

B. Datasets

The biological networks are available at Github (https://github.com/gilles-didier/MolTi) and can be used to test the software. A markdown file to generate updated version of the biological networks is also available.

REFERENCES

1. N. Del-Toro, M. Dumousseau, S. Orchard, R. C. Jimenez, E. Galeota, G. Launay, J. Goll, K. Breuer, K. Ono, L. Salwinski, and H. Hermjakob, "A new reference implementation of the PSICQUIC web service." Nucleic acids research **41**, W601–6 (2013).
2. T. Rolland, M. Taşan, B. Charloteaux, S. Pevzner, Q. Zhong, N. Sahni, S. Yi, I. Lemmens, C. Fontanillo, R. Mosca, A. Kamburov, S. Ghiassian, X. Yang, L. Ghamsari, D. Balcha, B. Begg, P. Braun, M. Brehme, M. Broly, A.-R. Carvunis, D. Convery-Zupan, R. Corominas, J. Coulombe-Huntington, E. Dann, M. Dreze, A. Dricot, C. Fan, E. Franzosa, F. Gebreab, B. Gutierrez, M. Hardy, M. Jin, S. Kang, R. Kiros, G. Lin, K. Luck, A. MacWilliams, J. Menche, R. Murray, A. Palagi, M. Poulin, X. Rambout, J. Rasla, P. Reichert, V. Romero, E. Ruyssinck, J. Sahalie, A. Scholz, A. Shah, A. Sharma, Y. Shen, K. Spirohn, S. Tam, A. Tejeda, S. Trigg, J.-C. Twizere, K. Vega, J. Walsh, M. Cusick, Y. Xia, A.-L. Barabási, L. Iakoucheva, P. Aloy, J. DeLasRivas, J. Tavernier, M. Calderwood, D. Hill, T. Hao, F. Roth, and M. Vidal, "A Proteome-Scale Map of the Human Interactome Network," Cell **159**, 1212–1226 (2014).
3. A. Paz, Z. Brownstein, Y. Ber, S. Bialik, E. David, D. Sagir, I. Ulitsky, R. Elkon, A. Kimchi, K. B. Avraham, Y. Shiloh, and R. Shamir, "SPIKE: a database of highly curated human signaling pathways." Nucleic acids research **39**, D793–9 (2011).
4. M. Kanehisa, M. Araki, S. Goto, M. Hattori, M. Hirakawa, M. Itoh, T. Katayama, S. Kawashima, S. Okuda, T. Tokimatsu, and Y. Yamanishi, "KEGG for linking genomes to life and the environment." Nucleic Acids Research **36**, 480–484 (2008).
5. C. F. Schaefer, K. Anthony, S. Krupa, J. Buchoff, M. Day, T. Hannay, and K. H. Buetow, "PID: the Pathway Interaction Database." Nucleic acids research **37**, D674–9 (2009).
6. D. Croft, A. F. Mundo, R. Haw, M. Milacic, J. Weiser, G. Wu, M. Caudy, P. Garapati, M. Gillespie, M. R. Kamdar, B. Jassal, S. Jupe, L. Matthews, B. May, S. Palatnik, K. Rothfels, V. Shamovsky, H. Song, M. Williams, E. Birney, H. Hermjakob, L. Stein, and P. D'Eustachio, "The Reactome pathway knowledgebase." Nucleic acids research **42**, D472–7 (2014).
7. G. Sales, E. Calura, and C. Romualdi, "graphite: GRAPH Interaction from pathway Topological Environment. R package version 1.12.0." (2014).
8. A. Ruepp, B. Waegele, M. Lechner, B. Brauner, I. Dunger-Kaltenbach, G. Fobo, G. Frishman, C. Montrone, and H. W. Mewes, "CORUM: The comprehensive resource of mammalian protein complexes-2009," Nucleic Acids Research **38**, 497–501 (2009).
9. P. J. Mucha, T. Richardson, K. Macon, M. a. Porter, and J.-P. Onnela, "Community structure in time-dependent, multiscale, and multiplex networks." Science (New York, N.Y.) **328**, 876–8 (2010).
10. V. Blondel, J. Guillaume, R. Lambiotte, and E. Mech, "Fast unfolding of communities in large networks," J. Stat. Mech p. P10008 (2008).
